# Adrenal Crisis Masked as Septic Shock in a Patient With Opioid Use Disorder on Buprenorphine

**DOI:** 10.7759/cureus.41794

**Published:** 2023-07-12

**Authors:** Ashley M Ebersole, Lucas McKnight, Akshay Vijayaraman, Alissa Guarneri, Andrea E Bonny

**Affiliations:** 1 Adolescent Medicine, Nationwide Children’s Hospital, Columbus, USA; 2 Internal Medicine: Pediatrics, The Ohio State University Wexner Medical Center and Nationwide Children's Hospital, Columbus, USA; 3 Addiction Medicine, The Ohio State University Wexner Medical Center and Nationwide Children's Hospital, Columbus, USA; 4 Pulmonary/Critical Care, The Ohio State University Wexner Medical Center, Columbus, USA; 5 Endocrinology, UPMC (University of Pittsburgh Medical Center) Children's Hospital of Pittsburgh, Pittsburgh, USA

**Keywords:** substance use, buprenorphine, pediatric hospital medicine, internal medicine and pediatrics, internal medicine and endocrinology, critical care and hospital medicine, sepsis and shock physiology, opioid use, adrenal insuficciency, adolescent addiction

## Abstract

Opioid-induced adrenal insufficiency is a known side effect of chronic opioid use, but opioid-induced adrenal insufficiency related to chronic buprenorphine-naloxone therapy is less well-known.

We present a case of a patient with opioid use disorder on chronic buprenorphine-naloxone therapy admitted with presumed septic shock and found to be in an adrenal crisis. The patient presented to our hospital with a shock-like presentation, requiring vasopressors, intubation, empiric glucocorticoids, and antibiotics. As her steroids were weaned, she developed bradycardia and blood glucose in the 60s. A low- and high-dose cosyntropin stimulation test confirmed the presence of secondary adrenal insufficiency, presumed to be due to her chronic buprenorphine-naloxone use. She was discharged on maintenance hydrocortisone and continued buprenorphine-naloxone therapy.

With the high prevalence of opioid use disorder and the common need for medication for opioid use disorder, it is important that healthcare providers properly identify opioid-induced adrenal insufficiency in order to quickly and correctly diagnose and treat adrenal crises.

## Introduction

Opioid-induced adrenal insufficiency (OIAI) is a known side effect of chronic opioid use, present in 5-15% and as high as 29% of long-term opioid users [1-4]. Risk factors associated with the development of OIAI are not clear. Several studies have compared opioid dose (expressed as morphine equivalent dose or MED) to the duration of opioid use independently. MED as low as 5 mg daily for two years, or 180 mg daily for just one month, has shown evidence of OIAI [5,6]. Buprenorphine, a partial opioid agonist, is the only opioid approved by the Food and Drug Administration for office-based treatment of opioid use disorder. OIAI related to buprenorphine use is rare but may be increasing in prevalence [4,7,8]. We present a case of a patient with OUD on chronic buprenorphine-naloxone therapy admitted with presumed septic shock and found to be in an adrenal crisis.

## Case presentation

A 19-year-old female on maintenance medication for opioid use disorder (MOUD) for severe OUD, with a history of chronic hepatitis C, immune thrombocytopenic purpura, and bipolar disorder, developed dizziness and abdominal pain one day prior to presentation. Home medications included buprenorphine-naloxone 8-2 mg twice daily, escitalopram 20 mg daily, quetiapine 50 mg nightly, and trazodone 150mg nightly. She had followed up with the institutional office-based opioid treatment clinic [9] and had been on buprenorphine-naloxone for the past two years. She had a history of intermittent cannabis, cocaine, and methamphetamine use, with opioid lapses one month and then two days prior to presentation. Other than these two episodes of opioid use, she denied use and had negative urine drug screens since starting with our treatment program two years prior.

She initially presented to an outlying hospital with fever and tachycardia. Workup and management included intravenous fluid resuscitation, blood and urine cultures, chest X-ray, and chest computerized tomography (CT); all were unremarkable. She left against medical advice prior to receiving antibiotics. The following day, she presented to our emergency department with a fever of 102.4°F, tachycardia of 134, headache, abdominal pain, and hypotension, with a blood pressure of 68/30. Despite aggressive intravenous fluid resuscitation, she required a norepinephrine infusion and empiric stress-dose hydrocortisone (75 mg/m^2^/day) [10]. She was intubated for respiratory support. Empiric vancomycin, cefepime, and amikacin were given. Initial lab evaluation showed leukocytosis to 23.6 k/µL, acute kidney injury with a serum creatinine of 1.49 mg/dL, elevated lactate of 4.5 mmol/L, and coagulopathy with an international normalized ratio of 1.95 and fibrinogen less than 60 mg/dL. Her procalcitonin and C-reactive protein were markedly elevated to 81.4 ng/ml and 20 mg/dL, respectively (Table 1).

**Table 1:** Relevant lab and imaging findings BNP: B-type natriuretic peptide; LDH: lactate dehydrogenase; TTE: transthoracic echocardiogram; Ig: immunoglobulin; MPO: myeloperoxidase; ACTH: adrenocorticotropic hormone

Lab	Hospital Day	Result		Reference Range
Chemistry	0	Sodium: 141, Potassium: 4.2, Chloride: 110, Carbon Dioxide: 18, BUN: 24, Creatinine: 1.49, Glucose: 77		135 - 145 mmol/L, 3.7 - 5.3 mmol/L, 95 - 106 mmol/L, 24 - 35 mmol/L, 5 - 18 mg/dL, 0.5 - 1 mg/dL, 60 - 115 mg/dL
White blood cells	0	23.6		4.5 - 13.5 x 10^3^/µL
Lactate	0	4.5		0.5 - 2.2 mmol/L
International normalized ratio	0	1.95		
Fibrinogen	0	<60		170 - 410 mg/dL
Procalcitonin	0	81.4		<0.5 ng/mL
C-reactive protein	0	20.0		< 1.2 mg/dL
Quantitative D-dimer	0	<0.27		<0.50 µg {FEU}/mL
Echocardiogram, transthoracic	0	1. Echocardiographic bright area near coronary sinus seen in apical four-chamber view. 2. No structural heart abnormalities. 3. No valvular abnormalities. 4. Normal biventricular size and systolic function. 5. No pericardial effusion. 6. Limited evaluation due to poor echocardiographic windows; if clinical concern is high for endocarditis, would evaluate by transesophageal echocardiogram.		
Echocardiogram, transesophageal	0	1. The septal aspect of the Eustachian valve, near the coronary sinus os, appears prominent but otherwise normal. This corresponds to the bright area noted in the prior TTE. There does not appear to be any additional mass, irregular surface, or hypermobile portion that would be suspicious for thrombus/vegetation. 2. No intracardiac vegetation. 3. No valvular abnormalities. 4. The tricuspid valve is normal. 5. Intact atrial septum. 6. Normal biventricular size and systolic function. 7. There is a retro-aortic coronary with flow going from right to left, which is suggestive of a circumflex artery off the right coronary artery. 8. No pericardial effusion.		
Lipase	0	19		< 20 U/L
Urinalysis	0	Source: Foley specimen. Urine color: Amber; Appearance: Cloudy; Specific gravity: 1.014 PH; (U): 5.0; Protein: Negative Glucose: Negative Ketones: Negative Bilirubin: Negative Occult Blood: Trace Nitrite: Negative Urobilinogen: 4 Leukocyte Esterase: Negative RBC: <1; WBC: <1; Squamous Epithelial Cells: <1; Mucus: Present		1.007 – 1.030 4.5 – 8.0 Negative Negative Negative Negative Negative Negative < 1.1 mg/dL Negative 0-2/[HPF] <6/[HPF] /[HPF]
Blood culture	0	No growth after 5 days		
HIV 1 & 2 antibody/antigen screen	0	Negative		Negative
Hepatitis A antibody (total)	0	Positive		Negative
Hepatitis B surface antigen	0	Negative		Negative
Hepatitis B surface antibody, Quantitative	0	16.58		m[IU]/mL
Hepatitis C antibody	0	Positive		Negative
CT abdomen and pelvis with IV and oral contrast	1	Nonspecific periportal edema in the liver with gallbladder wall thickening. Focal fatty change in the left lobe of the liver. Minimal free fluid in the pelvis. No loculated/drainable fluid collections. Diffuse body wall edema. Bibasilar atelectasis with trace right pleural effusion.		
Glucose (point of care)	3	66		60 - 115 mg/dL
TSH	3	0.995		0.4 – 4.0 u[IU]/mL
Free T4	3	0.9		0.7 – 2.1 ng/dL
BNP	3	157.0		<100.0 pg/mL
Troponin I	3	<0.010		<0.029 ng/mL
US pelvis with Doppler	4	Small amount of anechoic free fluid in the low pelvis, otherwise normal pelvic ultrasound		
CT head without contrast	4	No intracranial hemorrhage or other acute CT abnormality.		
HCG, urine	4	Negative		Negative
RPR	5	Nonreactive		Nonreactive
Chlamydia/gonorrhea/trichomonas amplified probe panel	5	Not detected		Not detected
Hepatitis C by quantitative nucleic acid amplification	5	Not detected		Not detected
LDH	6	LDH		325 – 650 U/L
Rheumatoid factor	6	<13		<15 [IU]/mL
Haptoglobin	6	102		33 – 171 mg/dL
Complement activity	6	122		60 - 144
C3 complement	6	83		86 – 184 mg/dL
C4 complement	6	24		16 – 59 mg/dL
IgG/IgA/IgM/IgE panel	6	IgG 847, IgA 216, IgM 390, IgE 231		546 – 1,842 mg/dL 60 – 327 mg/dL 45 – 140 mg/dL 0 – 257
MPO antibodies	6	Negative		Negative
PR3 antibodies	6	Negative		Negative
Crithidia antibody (IFA) titer	6	<1:10		<1:10
Anti-neutrophil cytoplasmic antibody	6	Negative		Negative
Cryoglobulin, qualitative	6	Negative, 72 hours		Negative
Dilute Russell's viper venom	6	None detected		< 1.2
ENA (SSA, SSB)	6	Anti-SSA <20 Anti-SSB <20		<20 units <20 units
ENA (Sm, RNP)	6	Anti-SM <20 Anti-RNP <20		<20 units <20 units
Anti-cardiolipin antibodies (IgG & IgM)	6	IgG <9.4 IgM 18.1		0 – 15 [GPL’U] 0 – 12.5 [MPL’U]
Beta 2 glycoprotein I antibody	6	IgG <10 IgM <10		<= 20 SGU <= 20 SMU
MR chest/abdomen/pelvis angiography with contrast	7	1. No evidence of abnormal vascular wall thickening or enhancement to suggest vasculitis. No filling defects to suggest thrombus or embolism. 2. Moderate edema within the thoracic, abdominal, and pelvic wall subcutaneous tissues, in addition to small volume pleural effusion and ascites. These findings are suggestive of anasarca. 3. No focal airspace disease. The solid and hollow viscera of the abdomen and pelvis are grossly normal. No evidence of lymphadenopathy, consolidation, abscess, or mass.		
ACTH	7	12		6 – 48 pg/mL
Low- and high-dose ACTH stimulation test	7	Medication: Cortrosyn 1 mcg (1410), Cortisol #1: 5.1 mcg/dL (Time: 1410), Cortisol #2: 5.5 mcg/dL (Time: 1430), Cortisol #3: 8.1 mcg/dL (Time 1450), Cortisol #4: 6.6 mcg/dL (Time 1510), Medication: Cortrosyn 250 mcg (1600), Cortisol #1: 8.8 mcg/dL (Time 1620), Cortisol #2: 9.9 mcg/dL (Time 1640), Cortisol #3: 11.0 mcg/dL (Time 1700)		
TB Quantiferon Gold plus	8	Not detected	Not detected	

The patient received standard septic shock care with subsequent improvement. Further evaluation, including transthoracic echo followed by transesophageal echocardiogram, and abdominal and pelvic CT, was unremarkable (Table 1). As hemodynamic status improved, she was weaned off vasopressors and extubated on hospital day three. Stress-dose steroids were continued. She completed seven days of cefepime with notable improvement of fever and procalcitonin level. The leading diagnosis was culture-negative sepsis.

Due to her lack of improvement with standard septic shock therapy and negative infectious workup, she underwent comprehensive rheumatologic evaluation (Table 1); serologic testing was only remarkable for an antinuclear antibody (ANA) titer of 1:640. Magnetic resonance angiography of the chest, abdomen, and pelvis to evaluate for vasculitis was notable only for mild anasarca. Human immunodeficiency virus and tuberculosis testing were negative.

With attempted discontinuation of stress-dose steroids, she developed bradycardia to the 30s with blood glucose in the 60s. Endocrinology was consulted for concerns of adrenal insufficiency. Her steroids were held on hospital day six, and the following day she underwent low- and high-dose cosyntropin stimulation tests with peak cortisol levels of 8.1 and 11 ug/dL, respectively. Pre-stimulation test adrenocorticotropic hormone level was 12 µg/dL. Utilizing a standard cut-off of 15.5 μg/dL for cortisol levels, stimulation testing was considered failed. A diagnosis of secondary adrenal insufficiency was made, presumably due to buprenorphine-naloxone use. She was then started on maintenance hydrocortisone (11 mg/m^2^/day). Buprenorphine-naloxone was resumed on hospital day four, and she was discharged on hospital day ten.

## Discussion

We highlight a rare case of partial opioid agonist-induced OIAI presenting in an adrenal crisis. Taoran et al. (2020) conducted a retrospective study of 40 patients with OIAI between 2006 and 2018 at a single center and found that only one patient in this cohort had an adrenal crisis similar to our patient [3]. There are no published clinical trials or randomized clinical trials on OIAI [7]. There are few reported cases of adrenal crisis among patients with opioid use, the majority of which are among patients on chronic opioids for pain management [8,11-15]. Literature regarding OIAI in patients with OUD is further limited. Das et al. (2014) describe a case of a 35-year-old patient with OIAI secondary to chronic heroin use [16] and Rasheed et al. (1995) studied 50 patients using heroin and found that serum cortisol levels were decreased in the majority of patients, irrespective of age, a dose of heroin per day, and period of drug intake [17].

Opioids exert negative effects on the hypothalamic-pituitary-adrenal axis, acting mainly at the hypothalamic-pituitary level where they inhibit corticotropin-releasing hormone, thus decreasing adrenocorticotropic hormone release [18]. Biochemical evidence of adrenal insufficiency as demonstrated in this patient is common, but effective treatment is unclear.

Treatment options include opioid reduction or discontinuation, allowing for hypothalamic-pituitary-adrenal axis restoration. The time needed for hypothalamic-pituitary-adrenal axis recovery has not been established [19]. Tapering opioid substitution therapy may not be feasible, as it is associated with increased rates of relapse [11]. Gibb et al. describe symptomatic improvement in OIAI with maintenance glucocorticoids [4]. Evidence supporting the efficacy of glucocorticoid replacement with MOUD for treating OIAI is lacking [19].

The patient's drug of choice historically has been methamphetamine, at times likely with opioids (fentanyl) as an additive. Her most consistent dosing of opioids has been through her buprenorphine daily dosing. While the occasional fentanyl included with her methamphetamine could have contributed to her adrenal insufficiency, it is our opinion that the daily buprenorphine dosing is much more likely to be the etiology.

This case highlights the importance of considering an adrenal crisis in OUD patients presenting with shock. Severe OIAI can present with a shock-like picture while milder OIAI can mimic opioid withdrawal. Studies of non-endocrine providers show that less than 70% are aware of OIAI and only 9% could identify OIAI symptoms [20], which include fatigue, nausea, vomiting, weight loss, dizziness, and muscle aches. With the rising need for MOUD, it is important that healthcare providers properly identify OIAI in order to quickly and correctly diagnose and treat adrenal crises.

## Conclusions

Prescribed and illicit opioid use is common in the community. Many patients will continue to require MOUD as part of their treatment. It is therefore important that healthcare providers identify a potentially severe adverse reaction, OIAI, and potential crisis in order to prevent morbidity and mortality in this vulnerable population.
